# Autistic Traits and Psychosocial Predictors of Depressive Symptoms

**DOI:** 10.1007/s10803-024-06361-y

**Published:** 2024-05-11

**Authors:** Lorna Camus, Kirsty Jones, Emily O’Dowd, Bonnie Auyeung, Gnanathusharan Rajendran, Mary Elizabeth Stewart

**Affiliations:** 1 Psychology Department, Heriot-Watt University, Edinburgh, EH14 4AS UK; 2 Division of Psychology, Sociology and Education, Queen Margaret University, Edinburgh, EH21 6UU UK; 3 Psychology Department, University of Edinburgh, 7 George Square, Edinburgh, EH8 9JZ UK

**Keywords:** Autistic traits, Social self-efficacy, Social motivation, Loneliness, Depressive symptoms, Mediation analysis

## Abstract

Higher rates of depression and of depressed mood are associated with autistic traits, and both are associated with social interaction factors, such as social self-efficacy, social motivation and loneliness. This study examined whether these social factors explain the association between autistic traits and depression. 658 participants (527 women) completed an online survey with measures of autistic traits (AQ), social self-efficacy (Social Self-Efficacy Scale), social motivation (Social Striving Assessment Scale), loneliness (UCLA Loneliness Scale) and depressive symptoms (Beck Depression Inventory-II). A mediation analysis found the relationship between autistic traits and depressive symptoms was fully mediated by the other three factors (*β*[indirect] = .005, z = 2.63, *p* < .01; *β*[direct] = .05, z = 1.58, *p* > .05), forming a pathway from autistic traits, to social self-efficacy, to social motivation, to loneliness and finally to depressive symptoms. These results suggest that targeting social self-efficacy may break this pathway and disrupt this relationship. Interventions targeting supporting positive social interaction should be considered.

A range of evidence suggests that either being autistic or having a high level of autistic traits is a risk factor for depressive symptoms (Buck et al., 2014; Ghaziuddin et al., 2002; Hallett & Crompton, 2018; Mayes et al., 2011; Simonoff et al., 2008). It is therefore important to understand the robustness of this relationship and which factors may help explain this relationship. Psychosocial factors such as social self-efficacy, social motivation, and loneliness are known to be related to depression (Rosbrook & Whittingham, 2010; Vickerstaff et al., 2007; Wei et al., 2005). These factors differ both in autistic people and those with high levels of autistic traits (Dubey et al., 2015; Vickerstaff et al., 2007; Whitehouse et al., 2009). Consequently, they are potential candidates for mediating the relationship between autistic traits and depressive symptoms.

## Relationships Between Depression, Autism, and Autistic Traits

Autistic people when compared to non-autistic people were found to be significantly more likely to report a diagnosis of depression (Lever & Geurts, 2016; Rai et al., 2018a, 2018b). Moreover, when compared to non-autistic controls, autistic participants were significantly more likely to report a diagnosis of depression (Lever & Geurts, 2016; Rai et al., 2018a, 2018b). In a study of participants in the Avon Longitudinal Study of Parents and Children birth cohort in Bristol, United Kingdom, Rai et al. (2018b) reported 19.8% of the 4073 autistic participants in their cohort had received a diagnosis of depression between the ages of 18 and 27, compared to 6% of the 219,769 non-autistic participants in their cohort. Similarly, Lundström et al. (2011) found the risk of depression was 12 times greater in their autistic sample compared to their non-autistic sample. These results are replicated when assessing depressive symptoms in autistic samples compared to controls (Hill et al., 2004; Lever & Geurts, 2016; Rai et al., 2018a; Whitehouse et al., 2009). For instance, Lever and Geurts (2016) found that autistic people scored higher on measures of depressive symptoms than non-autistic people in their sample.

Observing patterns in those with high autistic traits can help provide information and improve our understanding regarding factors relating to mental health and well-being outcomes in both non-autistic and autistic populations (Landry & Chouinard, 2016; South et al., 2020). Studies of autistic traits complement research in autistic samples and provide us with avenues to support people whether they have an autism diagnosis or not. It is particularly important to understand the relationships between autistic traits and mental health given that there are barriers to diagnosis (South et al., 2020). Similar to the findings in autistic samples, autistic traits were associated with higher levels of depressive symptoms in both university student (Liss et al., 2008; Reed et al., 2016; Rosbrook & Whittingham, 2010; White et al., 2011) and adult general population samples (effect sizes range from 0.21 to 0.49, Fietz et al., 2018; Rai et al., 2018a). Autistic traits were also associated with higher levels of clinical depression (Lundström et al., 2011; Rai et al., 2018a). In a study of participants in the Study of Twin Adults: Genes and Environment, Sweden, Lundström et al. (2011) reported the risk of depression was 5 times greater for the high autistic traits group compared to the low autistic traits group.

Autistic traits have a robust and consistently moderate relationship with depressive symptoms (Lundström et al., 2011; Mayes et al., 2011), it is therefore important to understand which factors may mediate this relationship (Mazurek, 2014; Reed et al., 2016; Rosbrook & Whittingham, 2010).

## Social Self-Efficacy, Depression, Autism, and Autistic Traits

Social self-efficacy is a person’s belief and confidence in their ability to initiate and maintain social contact and friendships successfully (Gecas, 1989). Social self-efficacy can be conceptualised as a collection of social cognitive processes and beliefs about one’s social behaviours. Measures of the concept have often focused on the latter (e.g. the Scale of Perceived Social Self-Efficacy, Smith & Betz, 2000), while the cognitive aspects (such as social information processing, Grieve et al., 2014) are often missed. While there is a difference between self-perceived ability and actual skill, the former can have an effect on the latter and on mental well-being (Maciejewski et al., 2000; Wei et al., 2005). Research has shown that both autistic people and those with higher levels of autistic traits show differences in their levels of social self-efficacy (Camus et al., 2023; Rosbrook & Whittingham, 2010; Vickerstaff et al., 2007). For instance, self-evaluated social competence was lower in a sample of 22 autistic children compared to a neurotypical mean (Vickerstaff et al., 2007), and was negatively correlated with autistic traits in a study of 231 university students (Rosbrook & Whittingham, 2010). Social self-efficacy has also been related to mental health and depression, for instance, self-rated social self-efficacy was negatively correlated to depressive symptoms in a sample of 308 university students, a relationship which was mediated by loneliness (Wei et al., 2005). Camus et al. (2023) found that both in- and out- group social self-efficacy in autistic people was positively related to mental well-being, and that in group self-efficacy was higher than out-group self-efficacy.

These studies therefore show that self-efficacy is a factor which differs in autistic people compared to non-autistic people, is important in mental health and well-being, and that factors such as loneliness and social group may be important in these relationships.

## Social Motivation

Social motivation has traditionally been understood as a set of social cognitions and behaviours which motivate a person to engage in social endeavours. These include *social orienting, social seeking* and *social maintaining* (Chevallier et al., 2012b). *Social orienting* is underpinned by the prioritisation of social stimuli, leading to observable preferences for social signals such as faces (Fletcher-Watson et al., 2008; Ro et al., 2001; Salva et al., 2011). *Social seeking* (or reward processing, Keifer et al., 2019) covers the inherent reward associated with social interaction (Fehr & Camerer, 2007; Hayden et al., 2007; Rekers et al., 2011). Finally, *social maintaining* (or reward motivation (wanting), Keifer et al., 2019) includes behaviours seeking to establish, maintain and improve relationships (Barbaro & Dissanayake, 2007; Chevallier et al., 2012c; Izuma et al., 2011). Past research has suffered from a lack of conceptualisation regarding which aspects of social motivation were being investigated (Keifer et al., 2019). Measures of social motivation, in particular self-report questionnaires (such as the Social Responsiveness Scale, Constantino & Gruber, 2005, or the Dimensions of Mastery Questionnaire, Morgan et al., 2019), tend to include items pertaining to different aspects of social motivation without discriminating between them.

Traditionally, research has proposed all three mechanisms of social orienting, seeking and maintaining to be impaired in autistic people, with examples such as lack of eye contact (Osterling et al., 2002), small number of friendships and reduced pleasure in social situations (Chevallier et al., 2012a; Howlin et al., 2004) and less observable maintaining strategies (Barbaro & Dissanayake, 2007; Chevallier et al., 2012c; Izuma et al., 2011). This has been theorised as being foundational to the ‘social impairments’ observed in autism (Bottini, 2018; Chevallier et al., 2012b), forming the social motivation theory of autism. However, this theory relies on neurotypical assumptions of social conventions, interactions or behaviours. More recently the validity of claims supporting a lack of social motivation in autism has been questioned (Jaswal & Akhtar, 2019). For instance, criticisms of eye contact and gaze research have challenged the use of social orienting (as it stands) as a measure of social motivation in autistic people. Research has shown that eye contact can vary between cultures (Richman et al., 1992; Zhang et al., 2006) and that reduced eye contact can have cognitive benefits for both autistic and non-autistic people, irrespective of levels of social motivation (Doherty-Sneddon & Phelps, 2005; Klin et al., 2002). In our study, the focus is on participants’ social maintaining behaviour as an indication of social motivation.

Social motivation has implications for a person’s well-being, underpinning important social behaviours understood to be central to one’s healthy development and adjustment. Low social motivation has also been found to be related to higher parent-reported (but not self-reported) social anxiety (Swain et al., 2015) and higher cortisol/stress in social situations in autistic people (Corbett et al., 2014). Social motivation is therefore an important factor in relation to well-being.

## Loneliness

Loneliness is defined as a subjective negative feeling of being isolated from others and of lacking social contact (Hays & DiMatteo, 1987), and may result from an unmet need or desire to have friends, or from the awareness of a difference between actual and desired social status (Locke et al., 2010). Previous studies have found a consistent positive association between high autistic traits and loneliness in both autistic and non-autistic samples (Hedley et al., 2018a, 2018b; Jobe & Williams White, 2007; Locke et al., 2010; Mazurek, 2014; Reed et al., 2016). Hedley et al. (2018b) suggest that being autistic (or having higher autistic traits) increases the likelihood of experiencing loneliness, which in turn can increase one’s risk of depression. This is supported by Hedley et al. (2018a)’s findings, in which loneliness moderated the relationship between autistic traits and depression, and Reed et al. (2016)’s results where loneliness mediated the relationship between autistic traits and quality of life. These findings challenge the idea that autism or autistic traits cause poor quality of life or well-being, and instead suggest that it is associated psychosocial difficulties (such as loneliness) which impact well-being most.

## Rationale, Aims, and Hypotheses

**Fig. 1:**
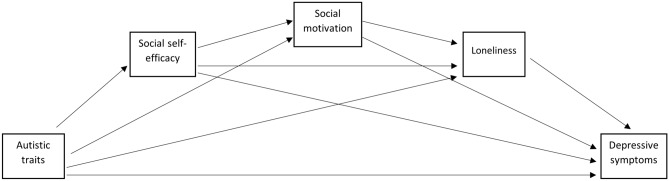
Theorised multiple serial mediation model of autistic traits as a predictor of depressive symptoms, mediated by social self-efficacy, social motivation and loneliness

While we know that the psychosocial factors of loneliness, social motivation, and social self-confidence are important in mental health, and that autistic traits are related to depression, we do not know if the pathway between autistic traits and depressive symptoms is through these psychosocial factors. Studies suggest that social self-efficacy is lower in both autistic people and those with high levels of autistic traits. A reduction in social self-efficacy may therefore affect a person’s confidence in engaging in social interactions, which may be perceived as a lack of social motivation. In turn, reduced social motivation (whether due to a lack of interest in social interactions as Chevallier et al. (2012c) propose, or due to a lack of confidence for example) may have an indirect effect on depression through loneliness. There is a distinction between being alone (social isolation) and being lonely (loneliness) (Age UK, 2022; Mazurek, 2014), and being alone (social isolation) may not be related to depressive symptoms. However, decreased social motivation could lead to the feeling of being isolated, and thereby lead to loneliness and depression. This suggests a pathway from autistic traits to depressive symptoms that is mediated by social self-efficacy, social motivation, and loneliness (see Fig. 1 for this theorised pathway).

The central aim of this study was to examine whether there exists a pathway connecting autistic traits to depression via the proposed psychosocial factors in autistic and non-autistic adults.

It was predicted that autistic traits and loneliness will be positively correlated with depression, while social self-efficacy and social motivation will be negatively correlated with depression; and the relationship between autistic traits and depression will be mediated by social self-efficacy, social motivation, and loneliness.

## Methods

### Participants

Participants were recruited through convenience sampling via two Scottish universities, social media, mailing lists and the Cambridge Autism Research Centre Database to complete the questionnaires in an online survey hosted on Qualtrics (2017).

Seven hundred and eighty-three participants completed the survey, 658 of which were included in the analyses—complete cases (no missing data) per measure ranged between 532 and 656. 123 participants were excluded due to missing data on two measures or more, and 2 participants were excluded as they were aged under 18. The sample consisted of university students and members of the general population, with an age range of 18–75 years old. There were 527 female participants, 119 males and 11 “others” (non-binary/transgender) (1 participant did not respond regarding their gender). Moreover, the sample was composed of 597 neurotypical participants and 61 autistic participants (self-reported).

### Measures

#### Autistic Traits

Autistic traits were measured using the Autism-Spectrum Quotient (AQ, Baron-Cohen et al., 2001), a 50-item, self-report scale. Items were scored on a 4-point Likert scale rather than the usual dichotomous scoring system to increase the scale’s sensitivity (Murray et al., 2016; Stewart et al., 2018), creating scores ranging from 50 to 200, with scores of 120 and above considered to reflect high autistic traits. The total AQ score was used in analyses. The AQ has shown satisfactory psychometrics (Stevenson & Hart, 2017). Internal consistency of the AQ scores was excellent in the current study ($\alpha$ = 0.935).

#### Social Self-Efficacy

Social self-efficacy was measured using the Social Self-Efficacy Scale (SSES, Grieve et al., 2014), a measure of a person’s perception of their ability to socialise. Participants are asked to rate how confident they are in 18 aspects of social interactions (e.g. “predict other people’s behaviour”). Ratings are made on a 5-point scale ranging from “not at all confident” to “very confident”, resulting in scores ranging from 18 to 90. Psychometrics for the SSES were found to be satisfactory (Grieve et al., 2014). Internal consistency of the SSES was excellent in this study ($\alpha$ = 0.956).

#### Social Motivation

Social motivation was measured using an adaptation of the Strivings Assessment Scales (Emmons, 1986). These scales measure a person’s striving towards self-created goals, and were adapted to assess striving towards social goals specifically. The adapted Social Striving Assessment Scale (SSAS) consists of 15 items looking at various personal aspects of social striving, such as emotions surrounding social interactions or commitment to social success. 13 questions were scored from 1 to 5, while 2 were required to be answered as a percentage probability, resulting in scores from 0 to 9. This produced scores ranging from 13 to 83. In the current study, the Cronbach alpha for the SSAS was found to be good ($\alpha$ = 0.746).

#### Loneliness

Loneliness was measured using the UCLA Loneliness Scale (UCLA, Russell, 1996). The 20-item measure includes questions regarding feelings surrounding interpersonal relationships (e.g. “how often do you feel part of a group of friends?”). Items are scored from 1 to 4 (“never” to “always”), 9 of which are reversed scored. This results in total scores ranging from 20 to 80. Psychometrics for the UCLA were satisfactory (Russell, 1996). The internal consistency of the UCLA in the current study was good ($\alpha$ = 0.759).

#### Depressive Symptoms

Depressive symptoms were measured using the Beck Depression Inventory—Second Edition (BDI-II, Beck et al., 1996). This is a 21-item measure including symptoms such as changes in appetite, sleep, feelings of sadness or pleasure. Each item is scored from 0 to 3, resulting in a range of scores from 0 to 63. The following cut-off scores were used for descriptive purposes: minimal symptoms, 0–13; mild symptoms, 14–19; moderate symptoms, 20–28; severe symptoms, 29–63. Scores of 29 and above are considered to indicate severe depression. The BDI-II has been shown to possess good psychometric properties in neurotypical (Beck et al., 1996) and autistic (Williams et al., 2021b) samples. The internal consistency of the BDI-II in the current study was excellent ($\alpha$ = 0.93).

### Procedure

Participants were provided with information on the study and were required to consent to participate prior to starting to complete the questionnaires. The project was granted ethical approval by University Ethics Committees. The survey collected demographic information (such as gender and date of birth), as well as medical information (autism diagnosis and psychiatric conditions) as well as the outlined questionnaires. The questionnaires pack took on average 25 min to complete. Following data collection, data were coded and anonymised prior to data analysis. The sample was collected in two waves (by KJ, EO’D) and collated and cleaned by LC.

### Analysis

Relationships between the social factors and depressive symptoms were examined via correlations. The potential pathway of these relationships was assessed via a serial mediation analysis.

## Results

### Descriptive Statistics

There were 47 cases of multivariate outliers (participants with a combination of unusual scores on at least two variables). However, upon individual inspection, all outliers were coherent within their own scores and within the range of scores of the sample, therefore they were not excluded.

Analyses revealed there were no significant gender differences for depressive symptoms, *t*(175.17) = 0.62, *p* = 0.54; and loneliness scores, *t*(145.57) = ‒0.99, *p* = 0.32. However, gender differences were found for autistic traits, social self-efficacy and social motivation. Males (*M* = 121, *SD* = 25) reported significantly higher scores on autistic traits than females (*M* = 109, *SD* = 21), *t*(155.33) = 5.02, *p* < 0.01. Females (*M* = 72, *SD* = 17) reported significantly higher social self-efficacy than males (*M* = 63, *SD* = 17), *t*(162.81) = ‒5.2, *p* < 0.01. Females (*M* = 54, *SD* = 10) reported significantly higher social motivation than males (*M* = 51, *SD* = 9), *t*(164.7) = ‒2.1, *p* = 0.04. As no gender differences were found for the outcome measure of depressive symptoms, gender was not included in the model.

Table 1 reports descriptive statistics for the sample. 113 participants reported having been or currently being depressed; 74 participants reported having had or currently having anxiety. Of these, 58 participants reported depression but not anxiety, 19 reported anxiety but not depression, and 55 reported both; for a total of 132 participants reporting having had or having depression and/or anxiety.

**Table 1:** Descriptive statistics

	n	Mean	sd	Min	Max
AQ	655	111.36	22.51	67	193
BDI-II	656	14.80	12.08	0	61
UCLA	596	52.47	7.68	24	74
SSES	604	69.98	17.86	20	105
SSAS	532	52.95	9.86	18	79

*AQ* Autism-Spectrum Quotient, *BDI-II* Beck Depression Inventory—Second Edition, *UCLA* UCLA Loneliness Scale, *SSES* Social Self-Efficacy Scale, *SSAS* Social Striving Assessment Scale

Of the 596 neurotypical participants who provided information on mental health diagnoses, 88 reported having been diagnosed with depression at some point in their lives (14.7%), and 56 reported having been diagnosed with anxiety at some point in their lives (9.4%). Of the 61 autistic participants, 25 reported having been diagnosed with depression at some point in their lives (41%), and 18 reported having been diagnosed with anxiety at some point in their lives (29.5%). Of the 658 participants who responded to the BDI-II, 361 reported minimal depressive symptoms (54.9%), 102 reported mild symptoms (15.5%), 96 reported moderate symptoms (14.6%), and 99 reported severe symptoms (15%).

### Mediation Analyses

All variables correlated with each other, except for loneliness and social self-efficacy (see Table 2 for full correlation matrix).

**Table 2:** Correlation matrix

	AQ	BDI-II	UCLA	SSES	SSAS
AQ		0.382***	0.183***	‒0.747***	‒0.521***
BDI-II	0.000		0.288***	‒0.422***	‒0.304***
UCLA	0.000	0.000		‒0.069	‒0.223***
SSES	0.000	0.000	1		0.496***
SSAS	0.000	0.000	0.000	0.000	

***p < .001

*Note: r* in upper half of table, corresponding *p* values in lower half

**Fig. 2:**
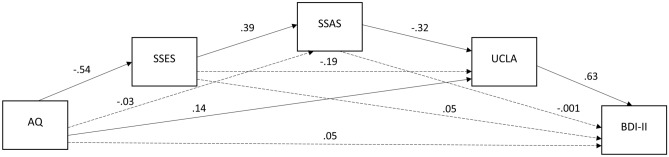
Multiple serial mediation model of autistic traits (AQ) as a predictor of depressive symptoms (BDI-II), mediated by social self-efficacy (SSES), social motivation (SSAS) and loneliness (UCLA). Dashed pathways are non-significant

As all factors of interest were related to BDI-II scores, we then assessed any potential mediation of the relationship between autistic traits and depressive symptoms. As hypothesised this effect was serially mediated by social self-efficacy scores, total social striving scores and loneliness scores. The indirect pathway of the effect of AQ on BDI-II via all three mediators was significant ($\beta$ [indirect] = 0.005, z = 2.62, *p* < 0.01). This pathway fully accounted for the overall impact of AQ on BDI-II with the direct effect being insignificant ($\beta$ [direct] = 0.05, z = 1.58, *p* > 0.05). Figure 2 illustrates this mediation.

## Discussion

The aim of this study was to identify a predictive pathway linking autistic traits to depression via a number of psychosocial factors. Depressive symptoms were associated with all four factors as predicted. In line with past research, correlation results indicated that depressive symptoms were positively related to higher levels of autistic traits and loneliness, while being negatively related to social motivation and social self-efficacy. As predicted, the relationship between autistic traits and depressive symptoms was mediated by social self-efficacy, social motivation and loneliness. The analysis confirmed that the three psychosocial variables serially mediated the relationship between autistic traits and depression. However, we did not expect that this relationship would be fully mediated by these factors as we found in this study. This challenges the notion that autistic traits are inevitably related to depression, but rather lends support to a social model of disability, where autistic people and those with high autistic traits may be socially excluded due to low social confidence, which may impact on psychological well-being. Additionally, social self-efficacy mediating the relationship between autistic traits and social motivation suggests that a perceived lack of interest in socialising may be due to a lack of confidence in one’s social skills (as Jobe & Williams White, 2007 suggest) rather than a lack of social motivation per se. Further research is needed to examine these relationships in more depth.

To our knowledge, this is the first study assessing the pathway between autistic traits and depressive symptoms via social self-efficacy, social motivation, and loneliness. This study’s results suggest that people with higher autistic traits are at an increased risk of depressive symptoms, and this is through a pathway of social self-efficacy to social motivation, to loneliness to depression. Depression is one of the leading causes of disability in the world (James et al., 2018) and this study has identified a potential factor to disrupt the pathway to depression for people with high autistic traits. Interventions targeting social self-efficacy may enhance other social factors and thereby reduce depressive symptoms. Given that depressive symptoms are higher in those with autistic traits (Fietz et al., 2018; Lundström et al., 2011; Rai et al., 2018a), this is an important finding which provides avenues for further research and support. The psychosocial factors in this study are malleable and provide target areas for enhancing mental health in those with high levels of autistic traits. For instance, a recent study showed that socially identifying with groups was positive for autistic people’s mental health (Maitland et al., 2021), therefore supports such as creating social groups could be a potential route. One possible avenue for future research would be to develop autistic peer support interventions and examine whether they provide mental health benefits for autistic people (Crompton et al., 2020).

This study has several limitations. First, the sample was obtained through convenience sampling and consisted of a large number of young university students, who represent a specific subset of the general population. While members of the general population also participated in this study, it will be important to replicate these results in a more varied sample. Secondly, the correlational design of this study limits the conclusions we can draw from our results. We cannot claim any precedence in the relationships nor can we conclude causal relationships from the current results. Further research is needed to replicate these results in longitudinal and experimental designs to make such conclusions. Finally, using autistic traits and depressive symptoms has limitations, as we cannot readily generalise our findings to autistic and clinically depressed populations. However the sample did include a) 9.2% percent of autistic people, and b) 45.1% percent of people who scored above the BDI-II cutoffs. It is of interest to note that correlations were similar between autistic and non-autistic samples. We know that autistic traits are highly related to depression, it is therefore of importance to understand the factors which affect this relationship. As people with high autistic traits and formally diagnosed autistic people can experience similar difficulties (Cassidy et al., 2020), future studies should aim to replicate these results in autistic samples. In particular, further research is needed to assess whether social self-efficacy predicts depressive symptoms and depression and whether interventions targeting these psychosocial factors reduce depression. Furthermore, considering there is a growing body of research suggesting autistic people feel more comfortable interacting with other autistic people (Crompton et al., 2020; Williams et al., 2021a), it may well be that people with high autistic traits have similar experiences. If that is the case, interventions targeting social self-efficacy based on the present results should take this into account.

## Conclusion

In conclusion, this study replicates previous findings showing relationships between autistic traits, social self-efficacy, social motivation, loneliness and depressive symptoms. It also provides new insights by suggesting that targeting social self-efficacy may provide mental health benefits in people with high autistic traits, and that a perceived lack of social motivation may be due in part to a lack of social self-efficacy (social confidence) in one’s social skills rather than lack of interest in socialisation. This study seems to indicate that the findings are similar within autistic people, however replication in autistic samples is needed. Further research should also assess whether social self-efficacy changes depending on social context (e.g. who you are interacting with). In addition, it would be of interest to determine how enhancing social self-efficacy would improve well-being in both autistic and non-autistic populations.
